# Cancer-associated adipocyte-derived G-CSF promotes breast cancer malignancy via Stat3 signaling

**DOI:** 10.1093/jmcb/mjaa016

**Published:** 2020-04-02

**Authors:** Li Liu, Yudong Wu, Cheng Zhang, Chong Zhou, Yining Li, Yi Zeng, Chunbo Zhang, Rong Li, Daya Luo, Lieliang Wang, Long Zhang, Shuo Tu, Huan Deng, Shiwen Luo, Ye-Guang Chen, Xiangyang Xiong, Xiaohua Yan

**Affiliations:** 1 Department of Biochemistry and Molecular Biology, School of Basic Medical Sciences, Nanchang University, Nanchang 330006, China; 2 Department of Breast Surgery, Jiangxi Provincial Cancer Hospital, Nanchang 330029, China; 3 School of Pharmacy, Nanchang University, Nanchang 330006, China; 4 School of Basic Medical Sciences, Nanchang University, Nanchang 330006, China; 5 Life Sciences Institute and Innovation Center for Cell Signaling Network, Zhejiang University, Hangzhou 310058, China; 6 Department of Pathology, The Fourth Affiliated Hospital of Nanchang University, Nanchang 330003, China; 7 Center for Experimental Medicine, The First Affiliated Hospital of Nanchang University, Nanchang 330006, China; 8 The State Key Laboratory of Membrane Biology, Tsinghua-Peking Center for Life Sciences, School of Life Sciences, Tsinghua University, Beijing 100084, China; 9 Institute of Biomedical Sciences, Nanchang University Medical College, Nanchang 330031, China; 10 Jiangxi Province Key Laboratory of Tumor Pathogens and Molecular Pathology, Nanchang University Medical College, Nanchang 330006, China

**Keywords:** cancer-associated adipocyte, G-CSF, triple-negative breast cancer (TNBC), epithelial–mesenchymal transition (EMT), migration and invasion

## Abstract

Adipocyte is the most predominant cell type in the tumor microenvironment of breast cancer and plays a pivotal role in cancer progression, yet the underlying mechanisms and functional mediators remain elusive. We isolated primary preadipocytes from mammary fat pads of human breast cancer patients and generated mature adipocytes and cancer-associated adipocytes (CAAs) *in vitro*. The CAAs exhibited significantly different gene expression profiles as assessed by transcriptome sequencing. One of the highly expressed genes in CAAs is granulocyte colony-stimulating factor (G-CSF). Treatment with recombinant human G-CSF protein or stable expression of human G-CSF in triple-negative breast cancer (TNBC) cell lines enhanced epithelial–mesenchymal transition, migration, and invasion of cancer cells, by activating Stat3. Accordantly, targeting G-CSF/Stat3 signaling with G-CSF-neutralizing antibody, a chemical inhibitor, or siRNAs for Stat3 could all abrogate CAA- or G-CSF-induced migration and invasion of breast cancer cells. The pro-invasive genes MMP2 and MMP9 were identified as target genes of G-CSF in TNBC cells. Furthermore, in human breast cancer tissues, elevated G-CSF expression in adipocytes is well correlated with activated Stat3 signal in cancer cells. Together, our results suggest a novel strategy to intervene with invasive breast cancers by targeting CAA-derived G-CSF.

## Introduction

Breast cancer is the most common cancer type in women worldwide (Bray et al., 2018). Invasion and dissemination are the major causes behind the high mortality rate of breast cancer. Tumor microenvironment, i.e. the stroma, plays a pivotal role in breast cancer malignant transformation (Bussard et al., 2016; Laplane et al., 2018). Among the multiple cell types in the stroma, adipocyte at a close distance to breast cancer is emerging to play an active role in cancer progression (Wang et al., 2012; Choi et al., 2018).

Both normal mammary tissue and breast cancer tissue are rich of adipose tissue, which is composed of adipocytes, preadipocytes, macrophages, fibroblasts, immune cells, and others (Tan et al., 2011; Zwick et al., 2018). Adipocyte is the dominant cell type in normal mammary tissues, indispensible for postnatal mammary gland development (Zwick et al., 2018). Although long been recognized to play a supportive role and to act as an energy source, the adipose tissue also functions as an important endocrine source, secreting a wide array of peptides including hormones, cytokines, and growth factors, which are collectively regarded as adipokines (Wang et al., 2012; Nieman et al., 2013; Choi et al., 2018; Zwick et al., 2018). Consistent with this, adipocyte-secreted leptin contributes to proliferation and migration of epithelial breast cancer cells, and is clinically associated with breast cancer malignancy (Olea-Flores et al., 2018). In addition, other adipocyte-derived secretory proteins, such as collagen VI and its cleavage derivative endotrophin, plasminogen activator inhibitor-1 (PAI-1), and insulin-like growth factor binding protein 2 (IGF-BP2), were also shown to enhance the proliferative, migratory, and invasive capabilities of breast cancer cells (Iyengar et al., 2005; Carter and Church, 2012; Park and Scherer, 2012; Wang et al., 2015). Intriguingly, it was found that adipocytes co-cultured with breast cancer cells would undergo apparent phenotypic changes, such as decreased lipid content, gain of fibroblast-like cell morphology, decreased expression levels of late adipocyte differentiation markers like peroxisome proliferator-activated receptor gamma (PPAR-γ) and CCAAT enhancer-binding protein alpha (C/EBPα), and enhanced expression levels of pro-inflammatory cytokines including interleukin-6 (IL-6), IL-1β, and others (Dirat et al., 2011; Wang et al., 2012). These adipocytes were designated as ‘cancer-associated adipocytes’ (CAAs), which are capable of promoting epithelial–mesenchymal transition (EMT), invasion, and metastasis of breast cancer cells (Dirat et al., 2011; Bochet et al., 2013; Picon-Ruiz et al., 2016; Choi et al., 2018). Furthermore, CAAs could also promote breast cancer malignant progression by rewiring the metabolic pathways in cancer cells (Huang et al., 2017; Wang et al., 2017).

The EMT program converts epithelial cells into mesenchymal cells, which would lose the apical-basal cell polarity and cell–cell junctions in epithelial cells (Lamouille et al., 2014; Ye and Weinberg, 2015). In addition, the transdifferentiated cells exhibit elevated expression levels of mesenchymal markers like N-cadherin, vimentin, fibronectin, and smooth muscle actin alpha (α-SMA) and gain a front-rear polarity, thereby obtaining a more migratory phenotype. It is generally accepted that EMT, even if a partial EMT as observed in breast cancer, could be a key driving force for cancer progression by facilitating cancer cell local invasion, intravasation, dissemination in bloods, and finally extravasation (Katsuno et al., 2013; Olea-Flores et al., 2018). Moreover, EMT also endows breast cancer cells with traits of cancer stem cell and drug resistance (Luo et al., 2015). Cancer cell EMT could be driven by paracrine signals from the microenvironment, such as TGF-β, Wnt, Notch-1, Sonic Hedgehog (Shh), and pro-inflammatory cytokines including tumor necrosis factor alpha (TNF-α) and IL-6 (Heldin et al., 2012; Ye and Weinberg, 2015). Interestingly, adipocytes and CAAs in breast cancer stroma have been shown to induce cancer cell EMT through soluble factors such as IL-6 and leptin, both of which activate Stat3 signaling and promote cancer cell migration and invasion (Wang et al., 2012; Olea-Flores et al., 2018).

Granulocyte colony-stimulating factor (G-CSF, CSF3) belongs to a small secreted glycoprotein family that also includes macrophage colony-stimulating factor (M-CSF, CSF1), granulocyte–macrophage colony-stimulating factor (GM-CSF, CSF2), and a multi-CSF, i.e. IL-3 (Metcalf, 2010; Hamilton et al., 2016). G-CSF initiates signal transduction by binding to the single-pass membrane receptor G-CSFR and activating the downstream cellular signaling molecules such as Stat3, in addition to some others like PI3K/Akt and Ras/ERK mitogen-activated protein kinase (MAPK). Given its roles in regulating neutrophil and other hemopoietic/myeloid cells, G-CSF signaling has been found elevated in and associated with myeloid leukemia (Touw and van de Geijn, 2007). In addition, G-CSF and its receptor were also expressed in some solid tumors including lung cancer, glioma, bladder cancer, colorectal cancer, melanoma, skin carcinoma, and also breast cancer (Aliper et al., 2014; Mouchemore et al., 2018). G-CSF can promote invasion and metastasis of breast cancer cells by regulating the activities of stromal cells including neutrophils, macrophages, and fibroblasts (Kowanetz et al., 2010; Orecchioni et al., 2013; Samineni et al., 2013; Benesch et al., 2015; Hollmen et al., 2015; Welte et al., 2016). In addition, breast cancer cells, especially triple-negative breast cancer (TNBC) cells, are capable of secreting G-CSF, which then acts through autocrine or paracrine to promote breast cancer malignant progression (Park et al., 2011; Swierczak et al., 2014; Benesch et al., 2015; Zhao et al., 2015; Mouchemore et al., 2018). However, how G-CSF is involved in the breast cancer–stroma interaction and promotes breast cancer malignancy is not fully understood.

The present study reveals that breast cancer-activated CAAs are a new source of G-CSF in the tumor microenvironment. CAAs and G-CSF in turn act on breast cancer cells to promote malignant transformation, including EMT, migration, and invasion. In consideration of the fact that, in clinical breast cancer tissue samples, enhanced G-CSF expression in adipocytes is well correlated with high p-Stat3 level in cancer cells, these results provide not only novel mechanisms for the bidirectional influences between breast cancer and adipocytes/CAAs, but also new evidence for targeting CAAs or G-CSF/Stat3 signaling in treating disseminating TNBC.

## Results

### CAAs enhance migration and invasion of TNBC cells

**Figure 1:**
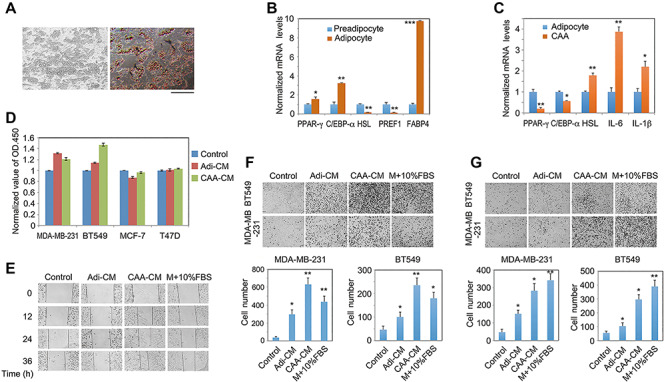
CAAs promote migration and invasion of TNBC cells. (**A**) Mature adipocytes were obtained by *in vitro* culture of primary human mammary preadipocytes with the adipogenic differentiation medium for 16 days. Cell morphology and lipid droplets were examined by phase-contrast microscopy and Oil Red O staining, respectively. Scale bar, 200 μm. (**B**) Confluent preadipocytes and mature adipocytes were subjected to total RNA extraction, reverse transcription, and real-time quantitative PCR (q-PCR). Gene expression levels were normalized to that of S18. All the q-PCR experiments were carried out similarly and in triplicate. (**C**) Mature adipocytes were co-cultured with or without MDA-MD-231 cells for 24 h in transwell, followed by RNA isolation and gene expression analysis as above. (**D**) Breast cancer cells were treated with Adi-CM, CAA-CM, or control DMEM medium (containing 0.2% FBS) for 48 h. Cell viability was determined by MTT assay. Data are expressed as the normalized value to that of control groups. (**E**) Cells were stimulated with Adi-CM, CAA-CM, or control DMEM, paralleled with a positive sample stimulated with 10% FBS-containing DMEM. Cell migration was monitored by following up the narrowing of the wound gap at the indicated time points under a phase-contrast microscope. (**F** and **G**) MDA-MD-231 and BT549 cells were cultured with Adi-CM, CAA-CM, or control DMEM in the upper chambers for 24 h, and then cell migration and invasion were assessed. Typical microscopic fields are shown, and cell number was quantified and presented as mean ± SD from at least three independent experiments. *P < 0.05, **P < 0.01, ***P < 0.001.

The resident mammary preadipocytes were successfully cultured *in vitro* (as described in Materials and methods) and exhibited a fibroblast-like morphology (Supplementary Figure S1A). Cytofluorometric analysis verified that they expressed the typical markers including CD44 and CD90, with the positive ratio of 98.46% and 92.37%, respectively (Supplementary Figure S1B). Mature adipocytes were generated from the preadipocytes by cultivation in the adipogenic differentiation medium for 16 days and characterized by the existence of lipid droplets as assessed by Oil Red O staining (Figure 1A). In addition, the expression levels of terminal differentiation markers, such as PPAR-γ, C/EBPα, and fatty acid-binding protein 4 (FABP4), were dramatically enhanced in mature adipocytes as compared with those in preadipocytes, whereas the expression levels of preadipocyte factor 1 (PREF1) and the hormone-sensitive lipase (HSL), two preadipocyte markers, were significantly reduced upon differentiation (Figure 1B), indicating that the *in vitro* differentiation system was able to yield mature adipocytes successfully.

Previous studies have demonstrated that co-culture with breast cancer cells would lead to activation of mature adipocytes into CAAs, which secrete a set of pro-inflammatory cytokines to promote malignant progression (Dirat et al., 2011; Drew et al., 2015; Picon-Ruiz et al., 2016). We co-cultured mature adipocytes with MDA-MB-231 breast cancer cells for 24 h and found that the expression levels of adipocyte makers, including PPAR-γ, C/EBPα, and FABP4, were reduced, while the expression levels of preadipocyte marker PREF1 and two typical pro-inflammatory cytokines IL-6 and IL-1β were significantly enhanced (Figure 1C), suggesting that the adipocytes had been converted to CAAs.

Next, we explored how CAAs may affect the cellular functions of breast cancer. Conditioned media (CMs) were generated from mature adipocytes (Adi-CM) and CAAs (CAA-CM), respectively. Then Adi-CM, CAA-CM, or control medium was used to treat various breast cancer cells but just exhibited limited effects on cancer cell viability or proliferation (Figure 1D; Supplementary Figure S2A). However, when compared with the control group, Adi-CM was able to promote wound healing efficacy of MDA-MB-231 cells to a certain degree, whereas CAA-CM promoted the process dramatically (Figure 1E). Similar results were obtained in testing the migratory capabilities of two TNBC cell lines MDA-MB-231 and BT549 by transwell experiments (Figure 1F). Furthermore, invasion assay with transwells also showed that Adi-CM and CAA-CM were capable of enhancing the invasive ability of the two TNBC cell lines (Figure 1G). However, the effects of Adi-CM or CAA-CM on migration or invasion of estrogen receptor (ER)-positive breast cancer cells were limited, such as MCF-7 and T47D cells (data not shown). Together, these results suggest that secreting factors are involved in CAA-induced migration and invasion of TNBC cells.

### Identification of G-CSF in CAAs by transcriptome and secretome profiling

**Figure 2:**
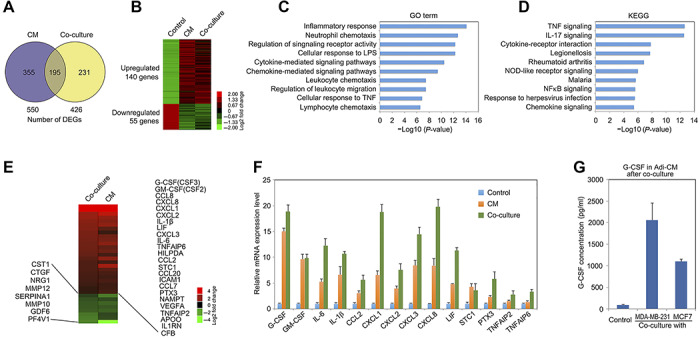
Transcriptome and secretome profiling of CAAs by RNA-Seq. (**A**) CAAs were generated by treatment with MDA-MB-231 cell-derived CM or co-cultured with MDA-MB-231 cells for 24 h, respectively. Then, CAAs and the untreated mature adipocytes were subjected to total RNA extraction and RNA-Seq. Gene expression level alterations with a fold change of >1.5 for upregulated genes and <0.6 for downregulated genes and with a *P*-value <0.05 were regarded as biologically significant. (**B**) Heat map classification of the 195 common DEGs from both CM-generated and co-culture-induced CAAs groups. Color-encoded relative gene expression levels are expressed in log2 scale. (**C** and **D**) GO analysis (**C**) and KEGG pathway enrichment (**D**) of the 195 DEGs. The top 10 GO terms or pathways were presented, respectively. (**E**) Heat map presentation of DEGs encoding secretory proteins among the common DEGs. Color-encoded relative gene expression levels are expressed in log2 scale. (**F**) Gene expression level detection for CAAs induced by breast cancer CM treatment or co-culture with MDA-MB-231 cells, along with control adipocytes, by RNA purification and q-PCR. (**G**) After co-culture of breast cancer cells and mature adipocytes in transwell for 36 h, breast cancer cells in the upper chambers were removed. Adipocytes and CAAs were further cultured with serum-free DMEM for another 24 h, and then G-CSF in the medium was quantified by ELISA.

To clarify gene expression alterations in breast cancer-mediated CAAs and to identify the possible functional soluble factors secreted by CAAs, mature adipocytes were treated with MDA-MB-231 cell-derived CM (CM group), co-cultured with MDA-MB-231 in transwell (Co-culture group), or cultured using control medium (Control group). After 24 h treatment or culture, the three groups of adipocytes/CAAs were subjected to total RNA isolation followed by transcriptome sequencing (RNA-Seq). Using >1.5-fold change for upregulation and <0.6-fold change for downregulation as cutoffs (Wang et al., 2018; Yan et al., 2018a), 550 and 426 differentially expressed genes (DEGs) were identified in CM- and co-culture-generated CAAs, respectively, in comparison to control adipocytes. Importantly, 195 genes were found overlapping in the two sets of DEGs (Figure 2A). As shown in the heat map (Figure 2B) and listed in Supplementary Table S1, 140 of the overlapping DEGs were upregulated, whereas 55 were downregulated. Interestingly, gene ontology (GO) term analysis showed that genes relevant to inflammatory response, chemotaxis, and chemokine signaling were highly enriched (Figure 2C). In Kyoto Encyclopedia of Genes and Genomes (KEGG) analysis, the most enriched signaling pathways include TNF-α signaling, NF-κB signaling, chemokine signaling, and others that are highly connected with inflammation and cell migration (Figure 2D).

Considering that soluble factors play a pivotal role in the functional crosstalk between breast cancer cells and adipocytes, we paid special attention to breast cancer-mediated secretome alteration in CAAs. As a result, 24 DEGs encoding secretory factors were found upregulated in both the CM group and the co-culture group, including some known pro-inflammatory genes such as IL-6 and IL-1β (Figure 2E). In addition, 8 downregulated DEGs were also identified (Figure 2E). Next, we verified by q-PCR that expression levels of these genes in adipocytes could be altered by MDA-MB-231 cell-derived CM or co-culture (Figure 2F). Furthermore, co-culture with another TNBC cell line BT549 or two ER-positive breast cancer cell lines MCF-7 and T47D also led to upregulation of those secretory factor-encoding genes in adipocytes (Supplementary Figure S3). Intriguingly, two of the CSF family members, G-CSF/CSF3 and GM-CSF/CSF2, were among the most highly upregulated genes (Figure 2E and F). Finally, we quantified by enzyme-linked immunosorbent assay (ELISA) the secreted protein level of G-CSF from CAAs induced by co-culture with MDA-MD-231 and MCF-7, respectively, and observed an enhanced secreting level of G-CSF when compared with adipocytes (Figure 2G). Furthermore, we noticed that stimulation of mature adipocytes with CM from different cancer cells, but not those from normal cell lines, could all increase G-CSF expression in adipocytes (Supplementary Figure S4), suggesting that cancer cell-mediated activation of adipocytes and their secretion of G-CSF could be a more generous event.

### G-CSF-neutralizing antibody inhibits CAA-mediated TNBC cell migration and invasion

**Figure 3:**
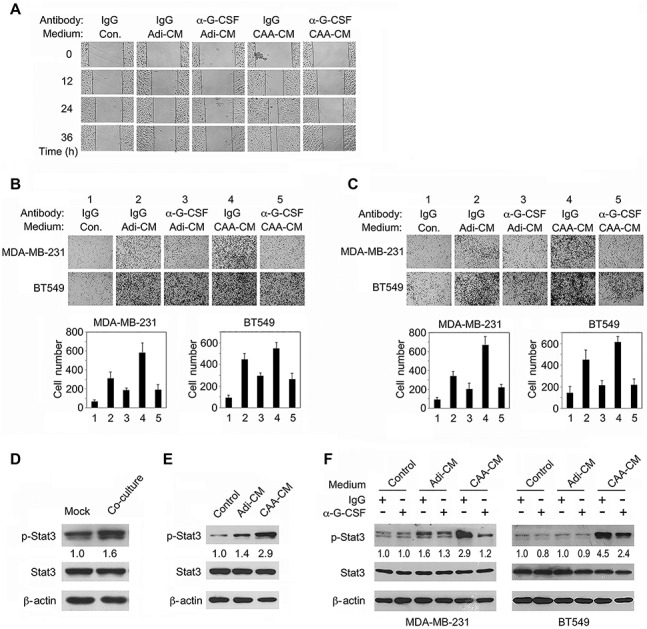
Targeting G-CSF with a specific neutralizing antibody inhibits CAA-induced TNBC cell migration and invasion. (**A**) MDA-MD-231cells cultured with Adi-CM, CAA-CM, or control DMEM were treated with G-CSF-neutralizing antibody or control IgG. Wound healing was monitored under a phase-contrast microscope. (**B** and **C**) MDA-MB-231 and BT549 cells cultured in different media were treated with G-CSF-neutralizing antibody or control IgG for 24 h, followed by transwell migration (**B**) and matrigel invasion (**C**) assays. (**D** and **E**) MDA-MB-231 cells co-cultured with adipocytes for 24 h (**D**) or treated with Adi-CM, CAA-CM, or control DMEM for 30 min (**E**) were lysed and analyzed for protein expression levels by western blotting. (**F**) MDA-MD-231 and BT549 cells cultured in CMs were treated with G-CSF-neutralizing antibody or control IgG for 15 min before western blotting. The phosphorylated Stat3 levels were quantified with Image J and normalized to that of Stat3. Typical microscopic fields and blots are shown and quantitative data are presented as mean ± SD from at least three independent experiments.

To address whether G-CSF mediates CAA-induced TNBC cell migration and invasion, we took use of a G-CSF-specific neutralizing antibody. As shown in Figure 3A, CAA-CM enhanced the migration of MDA-MB-231 cells in wound healing assay, whereas the Adi-CM had a minimal effect. However, the enhancing effects of Adi-CM and CAA-CM were blocked by G-CSF-neutralizing antibody. Similar results were also obtained in transwell migration and invasion assays (Figure 3B and C). As Stat3 is one of the major molecules mediating G-CSF signaling, we explored whether CAAs could activate Stat3 in TNBC cells. Indeed, co-culture with mature adipocytes led to enhanced Stat3 signaling activity in MDA-MB-231 cells, as determined by the protein level of tyrosine 705-phosphorylated Stat3 (Figure 3D). In accordance with it, Adi-CM or CAA-CM treatment of MDA-MB-231 cells could also activate Stat3 signaling, with CAA-CM having a more robust effect (Figure 3E). Importantly, G-CSF-neutralizing antibody was able to attenuate CAA-CM-induced Stat3 phosphorylation (Figure 3F). Together, these results implicate that CAA-secreted G-CSF plays a vital role in promoting migration and invasion of TNBC cells, by activating Stat3.

### Recombinant human G-CSF promotes TNBC cell migration and invasion by activating Stat3

**Figure 4:**
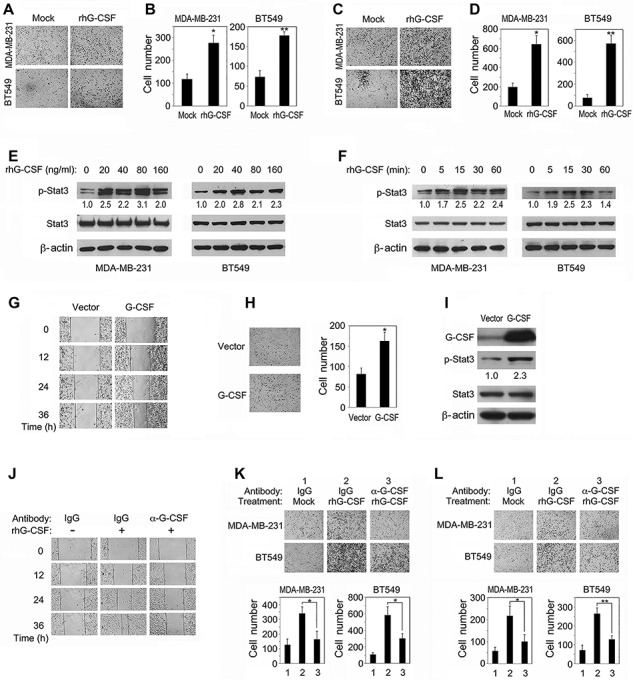
rhG-CSF promotes TNBC cell migration and invasion by activating Stat3. (**A**–**D**) MDA-MB-231 and BT549 cells cultured in the upper chambers in transwells were treated with or without 20 ng/ml rhG-CSF in DMEM containing 0.2% FBS for 24 h and subjected to migration (**A** and **B**) and transwell matrigel invasion (**C** and **D**) assays. (**E** and **F**) MDA-MD-231 or BT549 cells were treated with different concentrations of rhG-CSF in serum-free DMEM (**E**) or 20 ng/ml rhG-CSF for varying time periods (**F**) and examined for p-Stat3 protein levels. (**G**–**I**) Stable expression of G-CSF in MDA-MB-231 cells enhances their migratory (**G**) and invasive (**H**) capabilities and activates Stat3 signaling (**I**). (**J**–**L**) MDA-MD-231 cells treated with 20 ng/ml rhG-CSF in the presence of anti-G-CSF antibody or lgG were subjected to wound healing (**J**), transwell migration (**K**), and invasion (**L**) assays. Typical microscopic fields and blots are shown and quantitative data are presented as mean ± SD from at least three independent experiments. *P < 0.05, **P < 0.01.

We next assessed whether recombinant human G-CSF (rhG-CSF) could promote TNBC cell migration and invasion *in vitro*. MDA-MB-231 and BT549 cells were treated with 20 ng/ml rhG-CSF, respectively. In comparison to the untreated control groups, rhG-CSF treatment enhanced the migration and invasion of these cells in transwell assays (Figure 4A–D). However, rhG-CSF exhibited limited effects on breast cancer cell proliferation and viability (Supplementary Figure S2B and C), in accordance with the results regarding adipocytes/CAAs (Figure 1D). Next, we examined rhG-CSF-induced Stat3 activation in TNBC cells. Treatment with rhG-CSF of 20 ng/ml or higher concentrations for 15 min resulted in apparent activation of Stat3 (Figure 4E). As a support, time course examination of rhG-CSF stimulation showed that the p-Stat3 level culminated at 15 or 30 min (Figure 4F).

**Figure 5:**
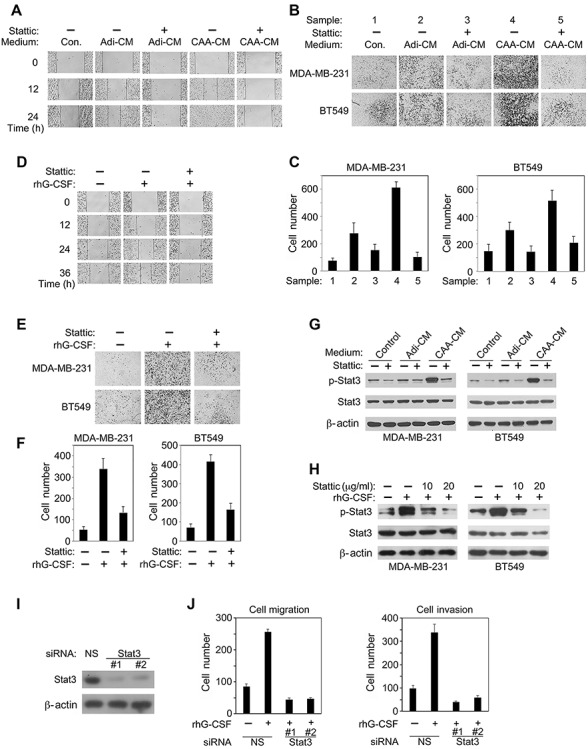
Targeting Stat3 by a small-molecule inhibitor abolishes CAA- and G-CSF-induced TNBC cell migration and invasion. (**A**–**C**) TNBC cells cultured in different media were treated with or without 20 μg/ml Stattic as indicated. Migration of MDA-MD-231 cells (**A**) and matrigel invasion of MDA-MD-231 or BT549 cells (**B** and **C**) were determined, respectively. (**D**–**F**) TNBC cells were treated with rhG-CSF (20 ng/ml) and Stattic (20 μg/ml) as indicated. Migration of MDA-MD-231 cells (**D**) and matrigel invasion of MDA-MD-231 or BT549 cells (**E** and **F**) were determined, respectively. (**G**) Cells cultured in different media were treated with or without 20 μg/ml Stattic for 15 min before western blotting for p-Stat3 protein levels. (**H**) Cells were treated with rhG-CSF and Stattic as indicated for 15 min before western blotting for p-Stat3 protein levels. (**I** and **J**) MDA-MB-231 cells transfected with nonspecific (NS) or Stat3-targeting siRNAs were analyzed by western blotting (**I**) or treated with 20 ng/ml rhG-CSF for 24 h and subjected to cell migration (left) and invasion (right) assays (**J**). Typical microscopic fields and blots are shown and quantitative data are presented as mean ± SD from at least three independent experiments.

To further validate the above observations, we established a MDA-MB-231 cell line that stably expresses human G-CSF. Indeed, the G-CSF-overexpressing cell line exhibited elevated migratory and invasive capabilities when compared with the vector-expressing cell line (Figure 4G and H). In line with it, stably expressed G-CSF was able to induce Stat3 phosphorylation (Figure 4I). Moreover, the neutralizing antibody was also capable of inhibiting rhG-CSF-induced migration and invasion of MDA-MB-231 and BT549 cells (Figure 4J–L), indicating that secreted G-CSF protein is able to promote migration and invasion of TNBC cells via Stat3 signaling.

### Targeting Stat3 abrogates CAA- and G-CSF-induced migration and invasion of TNBC cells

In order to determine whether Stat3 is a key downstream signaling molecule that mediates CAA- and G-CSF-induced malignancy of TNBC cells, we examined the effects of Stattic, a small-molecule inhibitor of Stat3 (Schust et al., 2006). Indeed, Stattic treatment abrogated Adi-CM- or CAA-CM-induced migration of MDA-MB-231 cells (Figure 5A), and invasion of both MDA-MB-231 cells and BT549 cells (Figure 5B and C). Stattic treatment could also lead to abolishment of rhG-CSF-induced migration of MDA-MB-231 cells and invasion of both MDA-MB-231 and BT549 cells (Figure 5D–F). In line with these observations, Stattic inhibited CAA-CM- or rhG-CSF-induced upregulation of p-Stat3 protein level (Figure 5G and H). Furthermore, we designed two siRNAs that could effectively knock down Stat3 gene expression (Figure 5I). Not surprisingly, compared with control groups, Stat3 gene depletion in MDA-MB-231 cells led to a dramatic reduction in rhG-CSF-induced cancer cell migration or invasion (Figure 5J). Together, these results indicated that Stat3 plays a crucial role in CAA- and G-CSF-induced migration of invasion of TNBC cells.

### G-CSF promotes EMT of breast cancer cells

**Figure 6:**
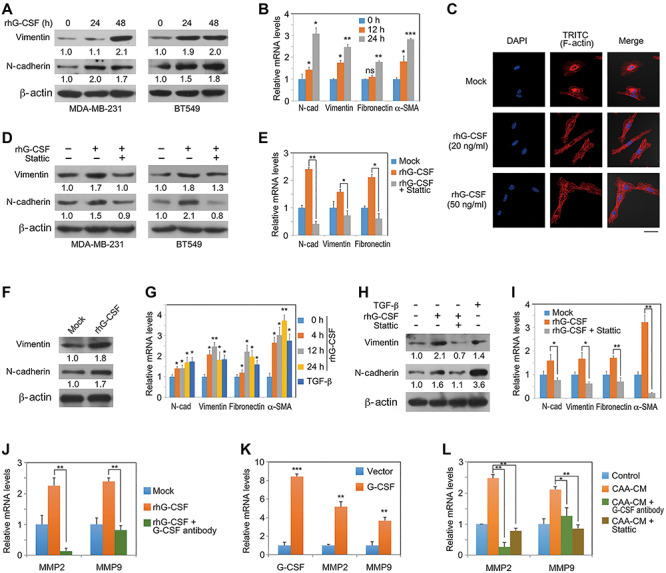
G-CSF promotes EMT of breast cancer cells. (**A** and **B**) TNBC cells were treated with or without 20 ng/ml rhG-CSF in 2% FBS-containing DMEM for the indicated time periods. Protein expression levels in MDA-MD-231 and BT549 cells (**A**) and mRNA levels in MDA-MB-231 cells (**B**) were detected, respectively. Protein expression levels were quantified with Image J and normalized to that of the loading control (β-actin). (**C**) MDA-MB-231 cells treated with G-CSF (20 or 50 ng/ml) for 24 h were stained with TRITC-labelled phalloidin. Scale bar, 40 μm. (**D** and **E**) MDA-MB-231 cells were treated with rhG-CSF (20 ng/ml) and Stattic (20 μg/ml) as indicated for 48 h. Protein (**D**) and mRNA (**E**) expression levels were determined. (**F** and **G**) NMuMG cells were treated with 20 ng/ml rhG-CSF for 48 h before western blotting (**F**) or for the indicated time periods before q-PCR (**G**). TGF-β1 ligand stimulation for 4 h served as a positive control in q-PCR analysis. (**H** and **I**) Stattic inhibits rhG-CSF-induced mesenchymal marker expression in NMuMG cells. (**J** and **K**) Gene expression levels in MDA-MB-231 cells treated with 20 ng/ml rhG-CSF and 1 μg/ml anti-G-CSF antibody as indicated for 4 h (**J**) or stably expressing G-CSF or control vector (**K**). (**L**) MDA-MD-231 cells cultured in CAA-CM or control DMEM were treated with G-CSF-neutralizing antibody or Stattic as indicated for 4 h before q-PCR analysis. Typical microscopic fields and blots are shown and quantitative data are presented as mean ± SD from at least three independent experiments. *P < 0.05, **P < 0.01, ***P < 0.001, ns indicates not significant.

Previous studies have demonstrated that mature adipocytes or CAAs were able to induce breast cancer cell EMT (Dirat et al., 2011; Park and Scherer, 2012; Lee et al., 2015; Yao-Borengasser et al., 2015; Gyamfi et al., 2018). However, whether G-CSF is a functional mediator in the process remains unknown. To answer it, we treated MDA-MB-231 cells and BT549 cells with 20 ng/ml rhG-CSF for 24 or 48 h. Western blotting results showed that the expression levels of mesenchymal markers, including N-cadherin and vimentin, were significantly upregulated (Figure 6A). rhG-CSF treatment of MDA-MB-231 cells also resulted in enhanced mRNA expression levels of N-cadherin, vimentin, fibronectin, and α-SMA (Figure 6B). Furthermore, rhG-CSF induced stress fiber formation of F-actin in MDA-MB-231 cells (Figure 6C). In addition, Stattic treatment abolished rhG-CSF-mediated upregulation of mesenchymal markers at both protein and mRNA levels (Figure 6D and E), indicating that G-CSF/Stat3 signaling is able to promote EMT of breast cancer cells.

**Figure 7:**
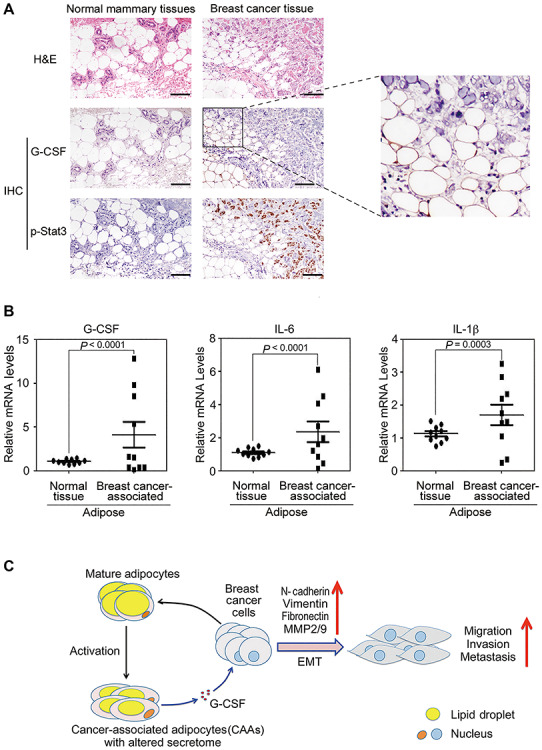
Clinical implications of G-CSF in adipocyte–breast cancer interaction. (**A**) Normal mammary tissues and breast cancer tissues were examined for G-CSF and p-Stat3 expression by H&E staining and IHC analysis. Scale bar, 200 μm. (**B**) Total RNAs were extracted from the adipose tissues. Gene expression levels were assessed by q-PCR and analyzed by GraphPad Prism5. (**C**) Schematic diagram shows the critical roles of G-CSF in adipocyte/CAA–breast cancer interplay. Breast cancer cells are able to convert adipocytes into CAAs that exhibit altered gene transcription pattern. In particular, G-CSF, which is highly expressed and secreted by CAAs, activates Stat3 signaling in TNBC cells. The G-CSF/Stat3 signaling then drives TNBC cell malignant progression by promoting EMT and invasion.

To further validate the above observations, we examined the effect of rhG-CSF on a normal mouse mammary epithelial cell line NMuMG. Similar to the results obtained in TNBC cell lines, rhG-CSF was able to promote the expression of mesenchymal markers in NMuMG cells at both protein and mRNA levels (Figure 6F–H). TGF-β was used as a positive control as it is a typical EMT inducer in various epithelial contexts (Figure 6G and H). However, rhG-CSF-mediated EMT of NMuMG cells was abolished by Stattic treatment (Figure 6H and I).

Finally, as matrix metallopeptidase 2 (MMP2) and MMP9 play an important role in EMT-associated migratory and invasive abilities (Katsuno et al., 2013), we asked whether the two genes could be regulated by G-CSF. Indeed, rhG-CSF was capable of increasing mRNA expression of both MMP2 and MMP9 in MDA-MB-231 cells, and this effect was abolished by G-CSF-neutralizing antibody (Figure 6J). Consistently, stably expression of human G-CSF in MDA-MB-231 cells also led to upregulation of MMP2 and MMP9 mRNA levels (Figure 6K). Moreover, G-CSF-neutralizing antibody and the Stat3 inhibitor Stattic were able to abrogate CAA-induced mRNA expression of MMP2 and MMP9, indicating that CAA-secreted G-CSF could induce expression of the pro-invasive genes MMP2 and MMP9 through Stat3 signaling.

### Clinical implications of G-CSF in adipocyte–breast cancer interaction

To further verify the potential significance of CAA-derived G-CSF in breast cancer development, we proceeded to analyze breast cancer tissues and normal mammary tissues obtained from human breast cancer patients. As shown in Figure 7A, hematoxylin and eosin (H&E) staining revealed that adipocytes in breast cancer tissues exhibited a smaller size and an irregular arrangement in comparison to those in normal mammary tissues, indicative of lipolysis in adipocytes and generation of CAAs in breast cancer (Dirat et al., 2011). In addition, immunohistochemistry (IHC) analysis showed that, although extremely low in normal mammary tissues, both G-CSF and p-Stat3 levels were high in breast cancer tissues, with elevated G-CSF level in adipocytes correlating well with strong p-Stat3 signal in cancer cells (Figure 7A). Importantly, gene expression analysis by q-PCR also supported that G-CSF is highly expressed in adipocytes from breast cancer tissues (Figure 7B). In addition to G-CSF, another two pro-inflammatory genes, IL-6 and IL-1β, also exhibited higher expression levels in breast cancer tissue-derived adipose (Figure 7B). These results suggest that G-CSF could be a *bona fide* functional mediator between CAAs and breast cancer in human breast cancer tissues.

## Discussion

The results obtained in the present study and from other laboratories have demonstrated that breast cancer cells and the paracancerous adipocytes have an intimate and bidirectional relationship (Tan et al., 2011; Wang et al., 2012; Nieman et al., 2013; Choi et al., 2018; Zwick et al., 2018). Breast cancer cells, either the TNBC or ER-positive cells, are able to convert mature adipocytes into CAAs, which have reduced lipid content due to lipolysis and enhanced expression levels of pro-inflammatory cytokines and chemokines (Dirat et al., 2011; Tan et al., 2011; Wang et al., 2012; Bochet et al., 2013). Importantly, although adipose tissue has been generally accepted as an endocrine organ, the last decade of investigations have uncovered its active role in promoting cancer initiation and progression, including breast cancer (Nieman et al., 2013; Choi et al., 2018; Zwick et al., 2018). In this regard, soluble factors have been discovered to play a pivotal role. Adipose stroma cell-derived IL-6, oncostatin M (OSM), and C-C motif chemokine ligand 2 (CCL2) have been shown to promote breast cancer cell proliferation, migration and invasion (Walter et al., 2009; Arendt et al., 2013; Lapeire et al., 2014). Furthermore, adipocyte- or CAA-secreted factors, including leptin, TNF-α, IL-6, IL-1β, IGFBP-2, LCN2, collagen VI, and others, have also been demonstrated to promote breast cancer cell malignant progression, such as EMT, invasion, and metastasis (Iyengar et al., 2003, 2005; Dirat et al., 2011; Drew et al., 2015; Wang et al., 2015; Olea-Flores et al., 2018). However, how breast cancer affects gene expression at the genome scale in adipocytes/CAAs and what new secretory factors may mediate the adipocyte–breast cancer interplay remain further elucidation. We performed RNA-Seq in this study to profile DEGs between mature adipocytes and CAAs, and genes relevant to inflammation and chemotaxis were the most highly enriched (Figure 2). Among them, G-CSF was identified as one of the most highly induced genes in CAAs. Our results indicate that adipocytes/CAAs are novel sources of G-CSF in the tumor stroma (Figures 2–4).

Functional investigations revealed that G-CSF is capable of promoting TNBC cell malignant transformation, such as EMT, migration, and invasion, by activating Stat3 signaling (Figures 3–6). Although mature adipocyte-generated CM could promote breast cancer cell migration and invasion to a certain degree, CM from CAAs exhibited a more dramatic effect (Figure 1). However, all of these promoting effects could be attenuated or abolished by treatments with the G-CSF-neutralizing antibody (Figure 4) or a Stat3-targeting small-molecule inhibitor (Figure 5), demonstrating that G-CSF is a crucial and functional mediator in the adipocyte/CAA–breast cancer crosstalk. In normal human mammary tissues, adipocyte is the most abundant cell type but encapsulated by basement membranes (Tan et al., 2011). However, when the invasive front of breast cancer disrupts the basement membranes, they will be able to contact with adipocytes directly (Tan et al., 2011; Wang et al., 2012; Choi et al., 2018). In this situation, soluble factors including G-CSF from adipocytes/CAAs would act on cancer cells to promote their EMT, migration, and invasion. In addition, our evidence also indicated that G-CSF could cooperate with other CAA-secreted factors, such as IL-6 and GM-CSF, to activate Stat3 signaling and promote breast cancer cell migration (Supplementary Figures S5 and S6).

Feedback and feed-forward regulatory loops play an important role in regulating signaling pathways and pathophysiological processes including cancer progression (Chang et al., 2014; Yan et al., 2018b). Interestingly, breast cancer-induced expression of G-CSF in CAAs is capable of promoting breast cancer cell EMT and motility, forming a feed-forward loop that amplifies adipocyte/CAA-mediated breast cancer progression. In support of it, G-CSF is highly expressed in breast cancer-associated adipose tissues in human patients (Figure 7B). Our results are also in line with previous reports that G-CSF is localized at the invasive front of breast tumors and is highly expressed in TNBC, compared with ER-positive breast cancer tissues or normal tissues, with bad clinical outcomes (Park et al., 2011; Aliper et al., 2014; Swierczak et al., 2014; Benesch et al., 2015; Zhao et al., 2015; Mouchemore et al., 2018).

Overweight and obesity put both adults and children at increased risk for poor health outcome (Picon-Ruiz et al., 2017). Obese individuals would accumulate large amounts of adipose tissues, which are closely connected with poor prognosis, especially in postmenopausal women. Intriguingly, adipocytes from obese adipose tissues and CAAs secrete a similar set of pro-inflammatory cytokines and chemokines, such as leptin, IL-6, IL-1β, and CXCLs (Khan et al., 2013; Picon-Ruiz et al., 2017). Although adipose stromal cell-derived GM-CSF has been shown involved in promoting breast cancer development (Reggiani et al., 2017), our results demonstrate that G-CSF is highly expressed in CAAs and in breast cancer-associated adipose tissues, conferring an invasive advantage on TNBC cells. Intriguingly, CM generated from other cancer cells could also reinforce the expression of G-CSF in adipocytes (Supplementary Figure S4), making it an interesting question as to whether G-CSF plays a role in obesity-associated carcinogensis and malignant development. In summary, our results provide novel insights into the mechanisms by which CAAs promote malignant progression of breast cancer and afford a promising choice for intervention of invasive breast cancer by targeting CAA-derived G-CSF.

## Materials and methods

### Reagents

rhG-CSF protein and TGF-β1 peptide were purchased from Peprotech and R&D Systems, respectively. The Stat3 inhibitor Stattic (HY-13818) was obtained from Med Chem Express (MCE). G-CSF-neutralizing antibody (ab9691) was purchased from Abcam. Anti-Stat3 (D1B2J), anti-Stat3 (p-Tyr705) (D3A7), anti-vimentin (D21H3), and anti-N-cadherin (D4R1H) antibodies were obtained from Cell Signaling Technology. Anti-β-actin (60008-1-Ig), anti-CD44 (ab216647), and anti-CD90 (66766-1-Ig) antibodies were from Proteintech. The nonspecific (NS) and two Stat3-specific siRNAs, targeting GGCGTCCAGTTCACTACTA and AGACCCGTCAACAAATTAA, respectively, were purchased from RiboBio.

### Cell lines and cell culture

The human TNBC cell lines MDA-MB-231 and BT549, the ER-positive breast cancer cell lines MCF-7 and T47D, and the mouse normal breast epithelial cell line NMuMG were purchased from the American Type Culture Collection (ATCC) or the Chinese National Infrastructure of Cell Line Resource. Among them, MDA-MB-231, MCF-7, T47D, and NMuMG cells were cultured in Dulbecco’s modified Eagle’s medium (DMEM, Corning) and BT549 cells were cultured in RPMI Medium 1640 (Corning). Both media were supplemented with 10% fetal bovine serum (FBS, Gibco), 100 U/ml penicillin, and 100 μg/ml streptomycin (Sigma-Aldrich). All cells were cultured in a humidified, 5% CO_2_-containing incubator at 37°C. Cell transfection was conducted with Lipofectamine 2000 (Invitrogen).

### Human mammary adipose tissue samples

Human normal mammary tissues and breast cancer tissues, both enriched with adipose, were obtained by tumorectomy from breast cancer patients from the Jiangxi Cancer Hospital, according to the guidelines of the Local Ethical Committee. All subjects provided their informed consent to participate in the study. The adipose tissue samples obtained were devoid of fibrotic tissues as assessed macroscopically and immediately used for collagenase digestion as described below.

### Isolation and characterization of human mammary resident preadipocytes

The preadipocytes were isolated from human mammary adipose tissues as described (Lapeire et al., 2014). Briefly, the freshly excised mammary adipose tissues from breast cancer patients were taken under sterile conditions, washed with cold phosphate-buffered saline (PBS), and then the blood vessels and fibrous tissues were carefully removed. The remaining products were minced, cut into small pieces, and then digested with collagenase (type I, Sigma-Aldrich) for 1 h at 37°C. The digestion products were filtered with a 100-μm cell sieve to remove debris and fibrous tissues, followed by centrifugation at 1000 rpm for 5 min. The pellets were suspended and the preadipocytes were cultured in DMEM/F12 with 10% FBS and penicillin/streptomycin antibiotics at 37°C in a humidified, 5% CO_2_-containing incubator. After three passages, the preadipocytes were characterized by cytofluorimetric analysis using anti-CD44 and anti-CD90 monoclonal antibodies.

### *In vitro* mature adipocyte generation and Oil Red O staining

Preadipocytes were cultured in DMEM/F12 with 10% FBS to >90% confluence in 6-well plates and then cultured in the adipogenic differentiation medium (HUXMD-90031, Cyagen) with 10% FBS for 15–16 days. The mature adipocytes were characterized by phase-contrast microscopy, Oil Red O staining, and q-PCR examination of adipocyte differentiation markers, such as PPAR-γ and C/EBPα. For Oil Red O staining, adipocytes generated by the *in vitro* differentiation system were fixed with 4% paraoxymethylene for 20 min, washed with PBS, and then stained with the Oil Red O solution for 30 min. After rinsing with PBS, the staining was checked under a microscope.

### Lentivirus production and stable cell line establishment

The full-length cDNA of human G-CSF-coding gene was inserted into the pL6.3-CMV-GFP-IRES-MCS lentivirus vector (Novobio Scientific). To produce defective lentivirus, HEK293FT cells were transfected using Lipofectamine 2000 with three plasmids, lentivirus-G-CSF or lentivirus-GFP, pCMVΔ8.9, and pM.G (VSVG), at the ratio of 2:1.5:1. The culture supernatants were collected at 48 h post transfection, and the viral particles were concentrated by centrifugation.

To establish G-CSF- or GFP-expressing stable cell lines, MDA-MB-231 cells were infected by the corresponsive lentivirus particles at a multiplicity of infection (MOI) of 50 pfu per cell, respectively. At 48 h post infection, the cells were treated with blasticidin (2 μg/ml), and the drug-resistant cells were pooled as stable cells, which were further maintained in DMEM in the presence of 10% FBS and blasticidin.

### Cell co-culture and CM production

Breast cancer cells and adipocytes/CAAs were co-cultured using a transwell co-culture system (0.4 μm pore size; Corning). Breast cancer cells were seeded in the upper layer of the transwell system, and adipocytes were seeded in the bottom layer. There was no direct contact between adipocytes and breast cancer cells. Adipocytes or cancer cells cultured alone under similar conditions served as controls.

Breast cancer cells or mature adipocytes were cultured in 6-well plates. After replacing with 0.2% FBS-containing DMEM and cultivation for another 24 h, the CM of breast cancer cells and Adi-CM were collected and filtered with 0.22-μm filters and stored at −80°C. To produce CAA-CM, mature adipocytes were co-cultured with breast cancer cells (normally MDA-MB-231 cells if not particularly indicated) in the transwell system for 36 h. Then the breast cancer cells in the upper layer were removed, and the activated adipocytes (CAAs) in the bottom layer were cultured in new 0.2% FBS-containing DMEM for another 24 h before the CAA-CM was collected.

### Transwell cell migration and invasion assays

A total of 1 × 10^5^ of breast cancer cells were seeded in the upper chamber of the transwell system with a pore size of 8.0 μm in the inserts (Corning), and mature adipocytes/CAAs were cultured in the bottom layer, with a control group without adipocytes. Another way is to stimulate the breast cancer cells in the upper chamber with Adi-CM, CAA-CM, or 0.2% FBS-containing control DMEM. After 24 h of co-culture or CM stimulation, breast cancer cells were fixed with 4% paraformaldehyde and stained using 1% crystal violet. The membranes were cleaned and air-dried, and the cell number on the membrane was counted in five random microscopic fields. Matrigel (BD Biosciences)-coated transwell inserts were utilized in invasion assays, which were then performed similarly as above.

### Wound-healing assay

Breast cancer cells were cultured in 12-well plates the day before. The confluent cell monolayer was wounded by scraping with a 20-μl pipette tip and then treated with Adi-CM, CAA-CM, or 0.2% FBS-containing control DMEM. Cell migration was monitored by following up the narrowing of the wound gap width in comparison to that of the start wound under a phase-contrast microscope at different time points. Experiments were performed in triplicate.

### RNA isolation, q-PCR, and western blotting

Total RNA extraction from cultured cells has been described previously (Yan et al., 2016, 2018a). To purify RNA from mammary adipose tissues, either normal or those surrounding cancer cells, 100 mg tissues were taken into ribozyme-free Eppendorf tubes. After addition of 1 ml TRIzol (TaKaRa) on ice, the adipose tissues were completely crushed with a tissue homogenizer and the tissue RNAs were extracted according to the manufacturer’s instruction.

For q-PCR analysis, 1 μg of RNA was reverse-transcribed using the PrimeScript RT Reagent Kit (TaKaRa). The resulting cDNA was used for q-PCR with the SYBR® Premix Ex Taq™ Kit (TaKaRa) in a StepOne Real-Time PCR Detection System (Life Technologies). Gene expression levels were normalized to that of S18 for adipocytes/CAAs or β-actin for breast cancer cells. Primers used for q-PCR are listed in Supplementary Table S2.

For western blotting, cells were lysed with lysis buffer (50 mM Tris-HCl, pH 7.5, 150 mM NaCl, 0.5% NP40, 1 mM EDTA, 10 mM NaF, 10 mM Na_4_P_2_O_7_, and 1 mM Na_3_VO_4_) containing protease inhibitors for 30 min at 4°C. After rotation at 4°C and centrifugation at 13000 rpm for 15 min at 4°C, the supernatants were removed to new Eppendorf tubes, denatured by adding loading buffer (200 mM Tris-HCl, pH 6.8, 8% SDS, 0.4% BPB, 40% glycerol, and 400 mM DTT) and boiling, and then subjected to SDA–PAGE gel and immunoblotting detection as described (Yan et al., 2016, 2018a). For gel quantification using Image J, the band intensity of total proteins was normalized to the loading control (β-actin) and that of phosphorylated proteins was normalized to the total proteins.

### RNA-Seq

The RNA samples from CAAs or control adipocytes were subjected to RNA-Seq with the Illumina HiSeq 2500 sequencer in BerryGenomics, Beijing. Transcriptome construction from RNA-Seq raw data and subsequent analyses were conducted similarly as described previously (Yan et al., 2018a), with updated databases or software as below. Briefly, raw single end reads were firstly trimmed of the first 13 bp from each end and mapped to the human genome (gencode.v25) with HISAT2 (v2.0.4). Gene expression level was estimated and normalized into an FPKM matrix using default parameters by StringTie (v1.3.0). DEGs were generated with Cuffdiff (v1.3.0) with *P*-value <0.05 and filtered by corresponding threshold. DEGs were subjected to GO term clustering and KEGG pathway enrichment by the NIH Database for Annotation, Visualization and Integrated Discovery (DAVID), with default parameters as reported, and a *P*-value <0.01 (adjusted by Benjamini) was considered statistically significant (Yan et al., 2018a).

### Cell proliferation assay

For MTT assay, breast cancer cells were seeded at 5000 cells per well in 96-well plates, in triplicate for each sample, and incubated for the indicated time periods. Then, 20 μl MTT (Solarbio) was added to each well, and the plates were incubated for 4 h at 37°C. After aspiration of the supernatants, 100 μl dimethyl sulfoxide (Solarbio) was added into each well, followed by incubation for 30 min at 37°C. The absorbance was measured at 450 nm in a SpectraMax® Paradigm® microplate reader (Molecular Devices). For cell counting, breast cancer cells were seeded into 24-well plates at 5000 cells per well. Cell number was counted after trypsinization and appropriate dilution at the indicated time points. The experiments were performed in triplicate.

### ELISA

Secretion of G-CSF by adipocytes was measured with ELISA development kit (EK1692, MultiSciences) according to the manufacturer’s protocol. Briefly, an aliquot (100 μl) of standard or the sample medium was added into different wells and then 50 μl biotin-labelled anti-G-CSF antibody was added to each well, followed by incubation for 2 h at room temperature. After that, 100 μl prepared streptavidin solution was added to each well and the plates were incubated for 45 min at room temperature. Then, 100 μl TMB One-Step Substrate Reagent was added to each well, followed by incubation for 30 min at room temperature. Finally, 100 μl Stop Solution was added and the sample absorbance was immediately measured at 450 nm using the SpectraMax® Paradigm® microplate reader, with the 570 or 630 nm correction wavelength.

### Cytofluorimetric analysis

A total of 1 × 10^6^ preadipocytes were trypsinized and fixed using 4% paraformaldehyde. After washing with PBS, cells were incubated for 3 h at 4°C with 10–20 mg/ml antibodies or the matched control isotypes. After a second wash with PBS that contains 0.5% BSA and 2% FBS, cells were incubated with secondary fluorescein-labelled IgG for 30 min at 4°C. Then the cells were analyzed in a FACScan flow cytometer (Becton Dickinson).

### H&E staining and IHC

The resident mammary adipose tissues were fixed in 10% neutral formalin for 48 h. After being trimmed of the oversized portion, the fixed tissue samples were placed in an embedding box, dehydrated with alcohol of different concentration, and then placed in xylene until being transparent. After that, the tissue block was embedded in paraffin and cut into thin slices on a microtome. H&E staining was carried out with the H&E staining solution (BaSo) according to the manufacturer’s introduction.

For IHC, sections of human breast cancer tissues and adjacent normal mammary tissues were treated with xylene and graded alcohol and then subjected to antigen retrieval in 0.01 M citrate buffer. Hydrogen peroxide was used for blockage. The sections were incubated with goat serum for 30 min and then with anti-G-CSF and anti-p-Stat3 antibodies overnight at 4°C. Subsequently, slides were incubated with biotin-linked secondary antibody and peroxidase-labelled streptavidin followed by a diaminobenzidine (substrate of peroxidase) revelation and counterstaining with Mayer’s hematoxylin. Slices were analyzed under a microscope.

### TRITC-phalloidin staining

After cultivation on coverslips and supernatant discarding, the breast cancer cells were washed with prewarmed PBS, fixed in 4% paraformaldehyde for 10 min, and permeabilized in 0.5% Triton X-100 for 5 min at room temperature. Then, 100 nM TRITC-phalloidin working solution (40734ES75, YE SEN) was added and the samples were incubated for 30 min at room temperature in the dark. After washing with PBS and mounting with DAPI-containing anti-fluorescent quencher (36308ES11, YE SEN), the staining was examined under a confocal microscope (ZEISS, LSM800).

### Statistical analysis

Quantitative data were presented as mean ± SD of at least three independent experiments. Data statistical analyses were performed by Student’s *t*-test or by one-way ANOVA for q-PCR, cell number quantification, and gene expression analysis in tissues. Differences were considered statistically significant when *P < 0.05, **P < 0.01, and ***P < 0.001.

## Funding

This work was supported by grants from the National Natural Science Foundation of China (NSFC; 31871378 and 31671460 to X.Y. and 81760509 to X.X.) and the Natural Science Foundation of Jiangxi Province of China (20171ACB21004 to X.Y. and 20181BAB205043 to X.X.).

**Conflict of interest:** none declared.

## Supplementary Material

mjaa016_Supplementary_Data
