# Supplementary material for: Cancer-associated adipocyte-derived G-CSF promotes breast cancer malignancy via Stat3 signaling
Source: J Mol Cell Biol. 2020 Apr 2;12(9):723–37. doi: 10.1093/jmcb/mjaa016 (PMC7749739; doi:10.1093/jmcb/mjaa016)
Supplement: mjaa016_Supplementary_Data [file mjaa016_supplementary_data.pdf]

Cancer-associated adipocytes-derived G-CSF promotes breast cancer malignancy via Stat3 signaling

Li Liu[1#], Yudong Wu[2#], Cheng Zhang[1], Chong Zhou[1], Yining Li[1], Yi Zeng[1], Chunbo Zhang[10], Rong Li[9], Daya Luo[1], Lieliang Wang[2], Long Zhang[8], Shuo Tu[1], Huan Deng[7], Shiwen Luo[6], Ye-Guang Chen[5], Xiangyang Xiong[1]*, Xiaohua Yan[1,3,4]*

[#]These authors contributed equally to this work.

*Correspondence: Xiangyang Xiong. Address: School of Basic Medical Sciences, Nanchang University, Nanchang 330031, Jiangxi, China; Email: xiangyangxiong@ncu.edu.cn;

*Correspondence: Xiaohua Yan. Address: School of Basic Medical Sciences, Nanchang University, Nanchang 330031, Jiangxi, China; Email: yanxiaohua@ncu.edu.cn.

List of Supplementary Materials

Figure S1: Cultivation and characterization of primary human mammary preadipocytes

Figure S2: CAAs and rhG-CSF have limited effects on growth or viability of breast cancer cells

Figure S3: Experimental verification of expression levels of secretory protein-encoding genes in CAAs

Figure S4: Cancer cell CM stimulation leads to enhanced expression of G-CSF in adipocytes

Figure S5: G-CSF cooperates with IL-6 to promote breast cancer cell migration

Figure S6: G-CSF cooperates with GM-CSF to promote breast cancer cell migration

Table S1: Common DEGs between co-culture- and breast cancer cell CM-induced CAAs

Table S2: q-PCR primers

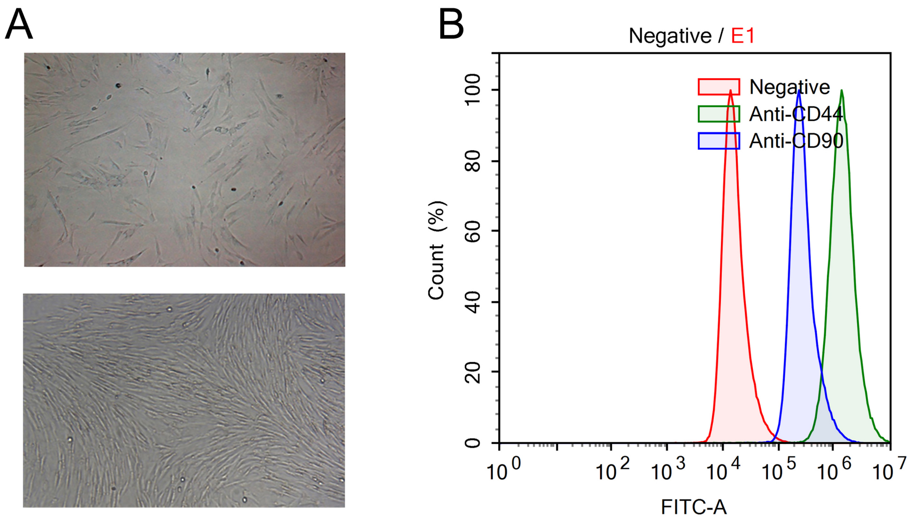

Figure S1. Cultivation and characterization of primary human mammary preadipocytes. **A**. Cultivation of primary human preadipocytes in vitro. Primary preadipocytes were obtained from mammary adipose tissues of human breast cancer patients by tumorectomy, as described in Methods. Typical phase-contrast pictures were presented to show the growth of preadipocytes of different confluency in dishes. **B**. Preadipocytes are positive for expression of CD44 and CD90. Gene expression was assessed by flow cytometric analyses using specific antibodies.

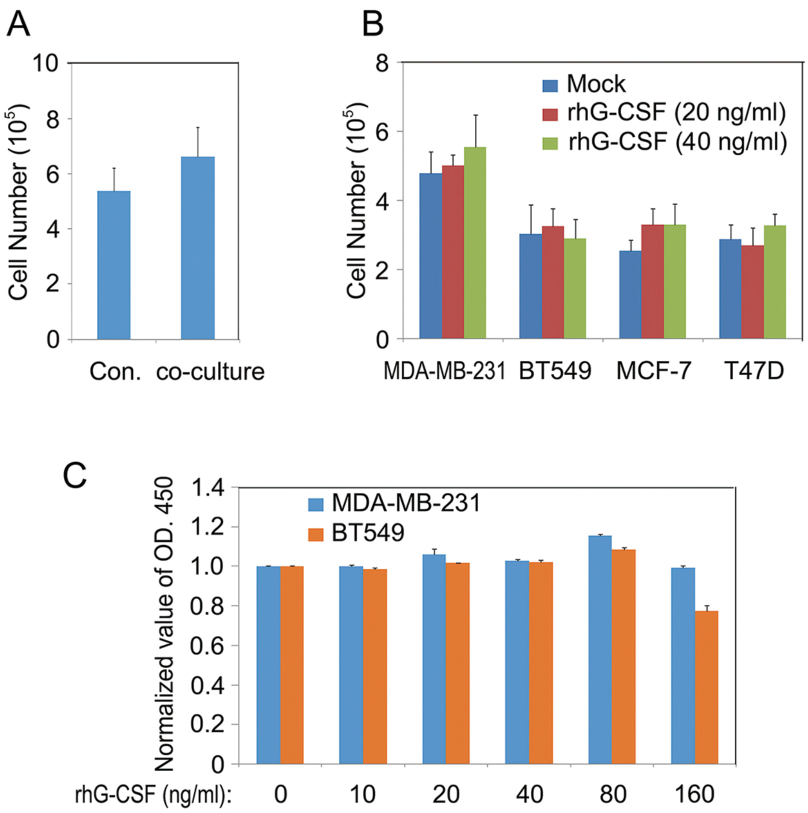

Figure S2. CAAs and rhG-CSF have limited effects on growth or viability of breast cancer cells. **A**. MDA-MD-231 cells were co-cultured with or without adipocytes. Cell growth was determined by cell counting. **B**. MDA-MD-231, BT549, MCF-7 and T47D cells were treated with different dose of rhG-CSF (20 ng/ml and 40 ng/ml) for 48 h respectively, followed by cell counting. **C**. rhG-CSF doesn't affect the viability of TNBC cells. MDA-MD-231 or BT549 cells were treated with rhG-CSF of varying dosage (0-160 ng/ml) for 48 h, followed by MTT measurement.

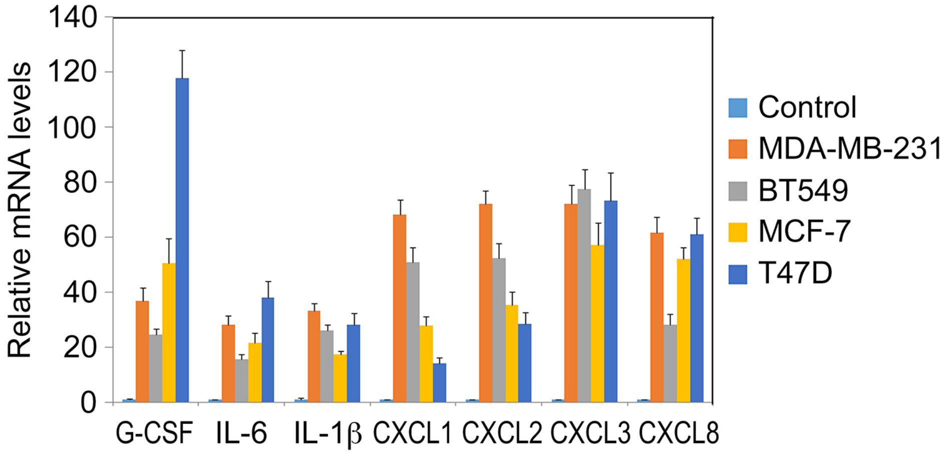

Figure S3. Experimental verification of expression levels of secretory protein-encoding genes in CAAs. Adipocytes were co-cultured with or without different breast cancer cell lines for 24 h. Gene expression levels in CAAs were determined by q-PCR.

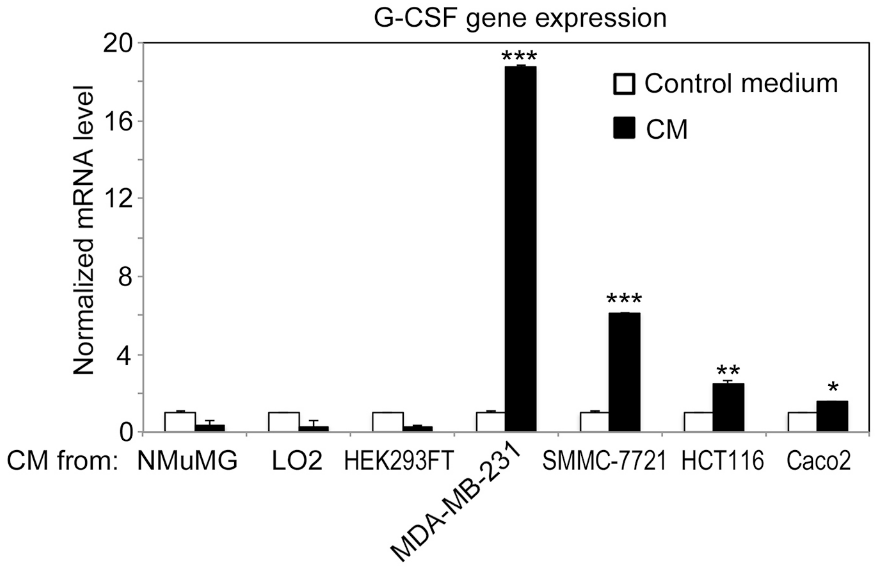

Figure S4. Cancer cell CM stimulation leads to enhanced expression of G-CSF in adipocytes. Conditioned media (CM) from different cell lines were used to stimulate mature adipocytes for 24 h, with DMEM treatment serving as the negative controls. Then, the G-CSF expression level in adipocytes was determined by RNA extraction and q-PCR analysis. ***, p value <0.001; **, p value <0.01; *, p value <0.05.

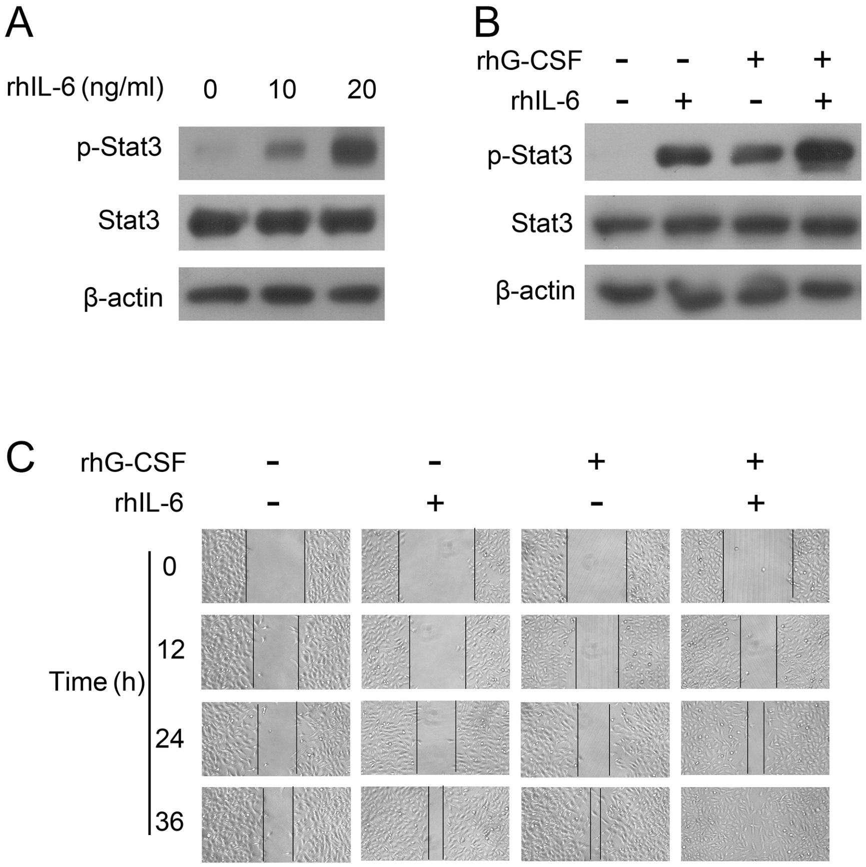

Figure S5. G-CSF cooperates with IL-6 to promote breast cancer cell migration. **A**. MDA-MB-231 cells were treated with different concentration of recombinant human (rh) IL-6 in serum-free DMEM for 15 min, followed by western blotting analysis using specific antibodies. **B**. MDA-MB-231 cells were treated with rhG-CSF (20 ng/ml) or rhIL-6 (20 ng/ml) as indicated for 15 min, and protein expression levels were determined by western blotting. **C**. MDA-MB-231 cells were treated with rhG-CSF (20 ng/ml) and rhIL-6 (20 ng/ml) as indicated in DMEM containing 1% FBS, and cell migration was assessed by wound healing.

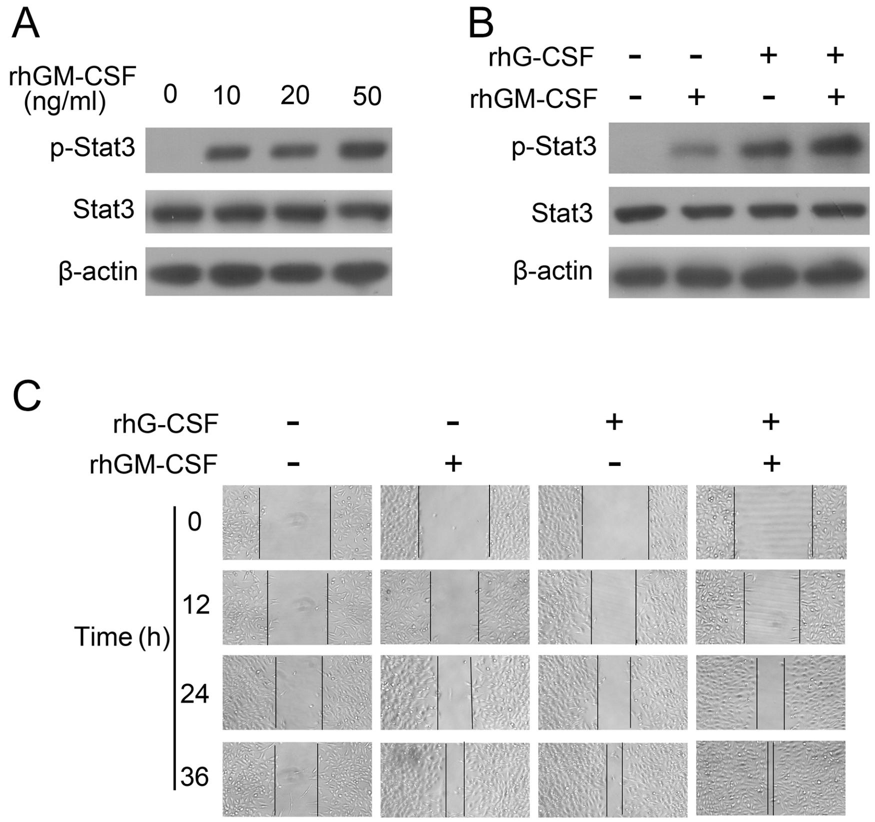

Figure S6. G-CSF cooperates with GM-CSF to promote breast cancer cell migration. A. MDA-MB-231 cells were treated with different dose of rhGM-CSF in serum-free DMEM for 15 min, followed by western blotting analysis. **B**. MDA-MB-231 cells were treated with rhG-CSF (20 ng/ml) or rhGM-CSF (20 ng/ml) as indicated for 15 min, and protein expression levels were determined by western blotting. **C**. MDA-MB-231 cells were treated with rhG-CSF (20 ng/ml) and rhGM-CSF (20 ng/ml) as indicated in DMEM containing 1% FBS, and cell migration was assessed by wound healing.

Common DEGs in co-culture- and CM-induced CAAs

	FPKM			Fold_change	
Gene	Control	Co-culture	CM	Co-culture/Control	CM/Control
CSF3	0.382985	9.41134	5.84187	24.5736456	15.2535168
CSF2	0.323597	5.19756	5.64403	16.0618927	17.4415941
THRSP	0.980233	6.19857	10.9439	6.32357321	11.1646026
RPL17-C18orf32	1.42139	6.54319	10.7359	4.60336871	7.55310582
RP11-3P17.5	1.11978	5.10782	5.37561	4.56144209	4.80058426
PTGS2	3.43857	15.5981	10.47	4.53621808	3.04488977
CCL8	2.03505	8.839	12.4003	4.34338548	6.09336854
CXCL8	68.1733	293.247	282.763	4.30147067	4.14769555
RP11-343C2.11	1.67929	6.66506	7.46364	3.96895719	4.44452734
CXCL1	103.19	386.901	373.158	3.74942665	3.61623893
CXCL2	6.67434	23.7375	27.1814	3.55652918	4.07251496
IL-1β	1.92003	6.76325	4.56424	3.5224763	2.37716163
SLC2A5	10.014	34.1191	36.664	3.4071371	3.66125942
HNRNPUL2-BSCL2	4.97577	16.4875	9.971	3.31355531	2.00391317
LIF	6.61111	21.4819	11.0653	3.24936993	1.67374878
CXCL3	9.65572	31.2331	28.1866	3.23467328	2.91915335
RPSAP54	2.41431	7.48662	4.69228	3.10094943	1.94352949
IL-6	25.4258	78.6578	80.9245	3.09360714	3.18276359
ZC3H12A	6.13156	18.8184	15.4778	3.06910925	2.52427659
TNFAIP6	17.2042	51.0312	65.5904	2.96620848	3.81245418
HILPDA	9.094	26.8498	23.6566	2.95248555	2.60133103
CCL2	64.4633	185.512	184.586	2.87778737	2.8634214
STC1	11.3651	30.5805	21.9076	2.69073442	1.92761108
PALM2	3.09267	8.21247	7.58084	2.65545669	2.45122004
BDKRB2	4.87126	12.8913	15.4174	2.64639791	3.164966
ADH1B	23.685	60.8182	71.455	2.56779495	3.01688562
POU2F2	2.95294	7.2232	8.56489	2.44611122	2.90045654
BDKRB1	4.56509	11.1184	9.16091	2.43553717	2.00673484
BNIP3	28.3136	68.1636	60.0442	2.40745695	2.1206845
PFKFB3	5.29248	12.5573	12.7745	2.37265115	2.41368934
CCL20	1.93549	4.58874	7.20978	2.37084279	3.72505054
RPLP0P2	2.73933	6.47757	6.03742	2.36465547	2.20397827
FTH1P7	39.8711	93.2182	80.2927	2.33799201	2.01379943
ZNF395	7.56369	17.3806	16.9576	2.29790635	2.2419747
ENO2	14.4103	32.7444	23.1866	2.27229477	1.60903756
SLC39A8	15.657	34.4028	45.6276	2.19728194	2.91420022
G0S2	187.765	406.754	450.616	2.16629542	2.39989286
NFKBIA	29.7199	64.1623	49.8238	2.15890536	1.67644831
DPYSL4	2.36068	5.08476	4.0797	2.15394289	1.72819019

	FPKM			Fold_change	
Gene	Control	Co-culture	CM	Co-culture/Control	CM/Control
ICAM1	18.2695	38.8943	34.1105	2.12891744	1.86707257
PLIN2	223.292	474.47	446.365	2.12487798	1.99902152
CCL7	3.69443	7.78686	17.8826	2.1077299	4.8404125
PTX3	322.222	676.287	757.617	2.09882227	2.35123705
GYS1	21.968	45.9	35.3201	2.08940187	1.60779559
SYNGR2	3.73204	7.63537	7.10869	2.04589202	1.9047729
MDP1	2.01007	4.1037	4.17587	2.04157137	2.07747339
DNAJC15	4.25007	8.46799	8.51679	1.99243409	2.00391317
NAMPT	68.5588	136.157	135.88	1.98599088	1.98194785
GPD1	10.0167	19.867	35.1233	1.98338397	3.50647149
PDK1	6.08212	12.0447	11.2696	1.98034529	1.85290511
DDIT4	69.8534	137.719	118.819	1.97154413	1.70098215
TNFAIP3	13.9875	27.5009	27.7898	1.96610858	1.98676743
BTG1	11.1118	21.793	18.4725	1.96124803	1.6624224
LYN	3.52499	6.86868	6.23198	1.94856762	1.76794329
VEGFA	27.8313	54.0532	50.4146	1.94217338	1.81143746
DTNA	5.95486	11.5447	17.9024	1.93870998	3.00634379
ZNF385A	4.47561	8.62849	8.2373	1.92789035	1.84048629
PRTFDC1	2.22364	4.26651	4.73526	1.91870112	2.12950778
ADGRD1	8.83367	16.8756	18.4744	1.91037783	2.09135794
DHRS3	11.4993	21.7821	25.3253	1.89421619	2.20234426
CTD-2369P2.8	3.72344	7.04828	6.16221	1.8929496	1.65498025
PTGES	49.552	93.0369	99.5386	1.87755998	2.00876668
ADM	91.6922	172.022	183.87	1.87607955	2.00528876
TREM1	2.13431	4.00338	4.70125	1.87572327	2.20269539
VLDLR	3.1861	5.97459	5.89359	1.87520328	1.84978039
NDRG1	55.4619	103.746	98.4554	1.87057654	1.77518945
NAMPTP1	8.74021	16.3213	16.8767	1.86737672	1.93092349
CIDEC	2.2082	4.12078	5.71399	1.86612548	2.58762759
TNFAIP2	23.3326	43.5292	46.7735	1.8655991	2.00464948
MVD	18.9968	35.106	47.2307	1.84799391	2.48624764
MARCH3	2.70116	4.97737	7.03676	1.84267674	2.60508419
PPFIA4	2.42145	4.46028	4.74498	1.8419846	1.95955763
TMEM37	6.72945	12.3443	16.7146	1.83436915	2.4838017
IRF1	9.82603	17.8786	16.0019	1.81951255	1.62851838
NFKBIZ	9.31547	16.6863	17.3551	1.79124416	1.86303916
AGAP1	5.41849	9.67395	10.6404	1.78535999	1.96371423
SLC2A1	17.8347	31.571	28.6375	1.770202	1.60571962
MELTF	2.98973	5.25118	4.88013	1.75640799	1.63230087
SLC2A6	9.51423	16.6885	16.0074	1.75405623	1.68246909
SPSB1	6.1652	10.783	9.33537	1.74901056	1.51420444

	FPKM			Fold_change	
Gene	Control	Co-culture	CM	Co-culture/Control	CM/Control
FAM110B	4.00769	6.99768	8.23852	1.74606105	2.05567187
CTD-3214H19.16	21.9094	38.1354	36.1055	1.74059915	1.64794832
APOO	4.73575	8.21124	8.22572	1.73388234	1.7369413
TXNIP	119.988	206.847	225.841	1.7238987	1.88219226
GRAMD1A	10.9475	18.8094	24.2359	1.71813572	2.21382318
ACSS2	15.6735	26.8614	38.2676	1.7138086	2.44155453
C8orf76	5.22956	8.9511	8.88495	1.7116349	1.69898604
RASD1	7.83552	13.4003	13.2269	1.71020231	1.68806915
SYNPO	5.02846	8.58609	8.31304	1.70749813	1.65319625
RP11-535A19.1	6.99581	11.9269	14.1032	1.7048585	2.0159502
NPAS2	8.96356	15.245	19.0108	1.70077229	2.1208903
SHMT1	3.74248	6.35971	6.45305	1.69933113	1.72427275
ZNF444	4.10271	6.94874	6.74267	1.69369723	1.64346872
FABP4	131.044	220.667	278.885	1.68391346	2.12817974
DRAM1	34.2907	57.5996	70.2434	1.67974473	2.04847459
IRF2BP1	4.01331	6.74089	6.02232	1.67963296	1.50058808
FYN	62.1834	103.986	107.432	1.67224125	1.72765841
RSF1	14.7521	24.6456	24.0698	1.67065634	1.63162328
TNFRSF1B	3.12064	5.19154	5.86651	1.66361316	1.87990532
C11orf96	8.87017	14.75	21.3789	1.66287647	2.41021192
SAA1	16.5677	27.4357	25.072	1.65597742	1.51330627
TRIM47	5.7616	9.44131	10.1126	1.63866052	1.75516419
FTH1P10	21.8253	35.7281	33.9362	1.63700644	1.55490668
A4GALT	15.1233	24.6749	23.5972	1.6315837	1.56032548
SLC29A4	2.63911	4.30541	5.19102	1.6313858	1.96696052
INSIG2	7.92783	12.9217	14.9811	1.62991305	1.88968007
TRIB3	10.0601	16.3833	16.4717	1.62854773	1.63733327
NFIB	15.1794	24.551	23.9336	1.61738821	1.57671291
SCARB1	10.727	17.3478	18.4689	1.61720885	1.72171578
PLD1	4.49106	7.26061	10.1503	1.61668208	2.26010531
SLC43A3	34.8653	56.2484	68.2752	1.61330365	1.95825277
ACOT1	3.83684	6.15523	6.69083	1.60424668	1.74384039
PNRC1	41.8017	66.8227	83.0846	1.59856344	1.98758836
PPP2R5B	3.61258	5.7728	6.26034	1.59797296	1.73292834
MPI	8.46368	13.5071	12.8911	1.59588755	1.52310246
LIPE	6.09337	9.71526	16.5721	1.59439932	2.71968582
DGAT2	28.4422	45.1754	46.203	1.58832154	1.62444962
PPL	3.11877	4.91884	9.01631	1.57717418	2.89098275
AVPI1	5.54625	8.74162	9.66014	1.57613051	1.74174328
NINJ1	23.8407	37.4621	37.69	1.57135266	1.58091193
CLNS1A	14.2786	22.4132	22.7595	1.56970451	1.59395511

	FPKM			Fold_change	
Gene	Control	Co-culture	CM	Co-culture/Control	CM/Control
TNFAIP8	10.3265	16.1924	20.0881	1.56804287	1.94529506
SUSD6	4.98496	7.81417	7.59179	1.56754841	1.52293672
HFE	4.00342	6.27395	6.53326	1.56714753	1.63191962
BHLHE41	6.5072	10.1912	11.0252	1.56614414	1.69430194
PLIN4	21.6324	33.8286	53.2628	1.56378915	2.46216936
ARRDC4	18.6543	29.096	32.44	1.55975129	1.73901214
NEGR1	6.32161	9.85999	10.5818	1.55972858	1.6739112
PGK1	103.708	161.386	173.889	1.55615525	1.6767063
C3orf18	2.97622	4.62855	4.55243	1.55517831	1.52960167
AMPD3	15.0462	23.1795	24.8413	1.54055907	1.6510033
TBC1D10A	4.42195	6.80914	7.70593	1.53985019	1.74265381
ALDOC	6.51024	10.0163	11.3097	1.53854538	1.73721823
PLA2G4C	5.12579	7.86376	9.34828	1.5341558	1.82377405
IL1RN	5.48005	8.36702	9.58274	1.52681366	1.74865902
CYP27A1	4.24479	6.45196	8.80636	1.51997121	2.07462416
LRIG1	8.21508	12.4527	14.7034	1.51583634	1.78980449
CFB	61.9474	93.8644	116.386	1.51522601	1.87879023
ABCB6	5.61959	8.49464	10.649	1.51161213	1.8949713
TMEM55A	6.06219	9.10201	11.8719	1.50144018	1.95834915
MYCBP	11.6567	6.97462	5.88549	0.59833526	0.50490155
IFIT1	20.2283	11.9053	5.83043	0.58854556	0.28823152
RGS4	7.59056	4.46092	3.28537	0.58769275	0.43282328
ITGA2	12.2657	7.1937	4.43328	0.58649146	0.36143798
CST1	13.4497	7.82013	4.63689	0.58143386	0.34475658
NEK10	6.56484	3.79977	3.33663	0.57880569	0.50825803
SLFN12	6.75851	3.88306	3.67675	0.57454428	0.54401714
BUB1B	9.64073	5.44883	5.21584	0.56518928	0.54102194
PLK4	6.45726	3.56733	2.63894	0.55245237	0.408679
MX1	14.4557	7.82475	2.08583	0.54129351	0.14429168
DNM3OS	13.7038	7.41453	7.72426	0.54105531	0.56365762
LPCAT4	8.18839	4.41548	4.56727	0.53923685	0.55777416
FKTN	6.4407	3.46238	3.81854	0.537578	0.59287752
RUNX2	4.38026	2.35046	2.40179	0.53660187	0.54832
GLRX2	12.7167	6.81979	7.58284	0.5362847	0.59628874
HERC6	9.90141	5.28506	2.78194	0.53376847	0.28096455
EEF1AKMT1	6.23655	3.31655	2.28167	0.53179238	0.36585402
RP11-244F12.3	4.82006	2.53475	2.87272	0.52587616	0.59599205
RP3-428L16.2	8.49278	4.43953	4.22951	0.52274128	0.49801117
ZNF789	5.08911	2.65702	2.74159	0.52209853	0.53871719
TSPAN2	23.5999	12.3049	10.9931	0.52139621	0.46580886
PI3	19.2654	10.024	5.06571	0.5203106	0.26294264

	FPKM			Fold_change	
Gene	Control	Co-culture	CM	Co-culture/Control	CM/Control
ATP1B1	6.75636	3.43573	3.03462	0.50851738	0.44915024
USP24	9.13652	4.60735	5.29915	0.50427863	0.57999686
MTCL1	7.14369	3.58522	3.79361	0.50187187	0.53104278
RP11-184M15.1	11.962	5.99027	5.92903	0.50077519	0.49565562
RP11-265D17.2	18.4501	9.2245	8.025	0.49996881	0.43495559
CTGF	151.774	75.8822	80.4063	0.49996534	0.52977475
CDH6	4.36087	2.16092	1.44083	0.49552509	0.33039872
MRVI1	4.52111	2.12899	2.47846	0.47089914	0.54819725
NRG1	12.1902	5.71811	5.11655	0.46907483	0.41972899
FLJ22447	5.70793	2.67279	1.85572	0.46825945	0.32511229
ISY1-RAB43	7.02009	3.26109	3.51611	0.46453848	0.50086406
MMP12	8.84192	4.07377	4.14307	0.46073529	0.46857114
TANGO6	4.89444	2.2328	2.40112	0.45619103	0.49057989
SNORD3B-2	7.69995	3.50703	2.57959	0.45546118	0.33501324
METTL18	4.3031	1.94828	1.96346	0.45276364	0.45628907
SERPINA1	6.70595	3.01009	2.91029	0.44887013	0.43398588
MFAP3L	6.8864	3.05254	3.97528	0.44327073	0.57726514
C2orf76	6.86054	2.9741	0.811033	0.43350784	0.11821705
ATR	7.28456	3.05781	3.02231	0.41976681	0.41489265
STARD4-AS1	6.4472	2.61238	3.66993	0.40519548	0.56922739
RP11-244F12.2	37.0363	14.9198	9.86904	0.40284032	0.26646907
EIF1AXP1	9.89942	3.95909	4.33026	0.39993016	0.43742573
SNORD3A	9.40494	3.7115	3.82379	0.39463204	0.40657122
GLMN	4.88912	1.9258	1.85195	0.39389418	0.37878996
SNORD3C	7.7138	2.8657	2.5695	0.37150409	0.33310294
MMP10	13.6095	4.87153	7.42939	0.35795	0.54589675
MIR503HG	20.3562	7.22241	9.60856	0.35480051	0.47202003
PCDH9	5.69929	1.97444	1.5263	0.34643582	0.26780411
GDF6	4.74885	1.61079	2.50649	0.3391934	0.52780978
DOCK10	18.2213	6.00239	8.58897	0.32941542	0.4713694
PF4V1	4.09671	1.31026	0.362558	0.31983284	0.08849992
TRAPPC5	18.3268	5.45979	9.84568	0.29791445	0.53722934
CTC-554D6.1	5.37828	0.19261	1.03528	0.03581257	0.19249328

Genes	q-PCR Primers (5'-3')
S18 (human)	Forward: ATCACCATTATGCAGAATCCACG
	Reverse: GACCTGGCTGTATTTTCCATCC
G-CSF (human)	Forward: GAGCAAGTGAGGAAGATCCAG
	Reverse: CAGCTTGTAGGTGGCACACA
MG-CSF (human)	Forward: GCGTCTCCTGAACCTGAGTA
	Reverse: GCGTCTCCTGAACCTGAGTA
CCL8 (human)	Forward: ATGCTGAAGCTCACACCCTT
	Reverse: CAAGTCCCTGAGGGCTGAAA
CXCL8 (human)	Forward: TTTTGCCAAGGAGTGCTAAAGA
	Reverse: AACCCTCTGCACCCAGTTTTC
CXCL1 (human)	Forward: TCCTGCATCCCCCATAGTTA
	Reverse: CTTCAGGAACAGCCACCAGT
CXCL2 (human)	Forward: GAAAGCTTGTCTCAACCCCG
	Reverse: TCTCCTAAGTGATGCTCAAACA
IL-1β (human)	Forward: AGCTACGAATCTCCGACCAC
	Reverse: CGTTATCCCATGTGTCGAAGAA
LIF (human)	Forward: CCAACGTGACGGACTTCCC
	Reverse: TACACGACTATGCGGTACAGC
CXCL3 (human)	Forward: GAGCACCAACTGACAGGAGA
	Reverse: AGTCCTTTCCAGCTGTCCCTA
IL-6 (human)	Forward: CCTGAACCTTCCAAAGATGGC
	Reverse: TTCACCAGGCAAGTCTCCTCA
TNFAIP6 (human)	Forward: TCTGTGCTGCTGGATGGATG
	Reverse: TCCTTTGCGTGTGGGTTGTA
CCL2 (human)	Forward: CAGCCAGATGCAATCAATGCC
	Reverse: TGGAATCCTGAACCCACTTCT
STC1 (human)	Forward: GTGGCGGCTCAAAACTCAG
	Reverse: GTGGAGCACCTCCGAATGG
CCL20 (human)	Forward: CAGCACTCCCAAAGAACTGG
	Reverse: CACTGACATCAAAGCAGCCA
PTX3 (human)	Forward: AGGCTTGAGTCTTTTAGTGCC
	Reverse: ATGGATTCCTCTTTGTGCCATAG

Genes	q-PCR Primers (5'-3')
TNFAIP2 (human)	Forward: GGCCAATGTGAGGGAGTTGAT
	Reverse: CCCGCTTTATCTGTGAGCCC
GAPDH (human)	Forward: ACAACTTTGGTATCGTGGAAGG
	Reverse: GCCATCACGCCACAGTTTC
N-cadherin (human)	Forward: TGCGGTACAGTGTAACTGGG
	Reverse: GAAACCGGGCTATCTGCTCG
Vimentin (human)	Forward: AGTCCACTGAGTACCGGAGAC
	Reverse: CATTTCACGCATCTGGCGTTC
Fibronectin (human)	Forward: AACCCTTCCACACCCCAATC
	Reverse: CCGGGAAGCTGAATACCGTT
α-SMA (human)	Forward: GGCATTCACGAGACCACCTAC
	Reverse: CGACATGACGTTGTTGGCATAC
PPAR-γ (human)	Forward: ACCAAAGTGCAATCAAAGTGGA
	Reverse: ATGAGGGAGTTGGAAGGCTCT
C/EBP-α (human)	Forward: TATAGGCTGGGCTTCCCCTT
	Reverse: AGCTTTCTGGTGTGACTCGG
HSL (human)	Forward: AGGAGCCAGCATTGAGACAAA
	Reverse: CGCAGGTGTTGATTCAGCTTC
PREF1 (human)	Forward: CTTTCGGCCACAGCACCTAT
	Reverse: TGTCATCCTCGCAGAATCCAT
FABP4 (human)	Forward: ACTGGGCCAGGAATTTGACG
	Reverse: CTCGTGGAAGTGACGCCTT
GAPDH (mouse)	Forward: CATCTTCCAGGAGCGAGACC
	Reverse: CTCGTGGTTCACACCCATCA
N-cadherin (mouse)	Forward: ATAGCCCGGTTTCACTTGAGA
	Reverse: CAGGCTTTGATCCCTCTGGA
Vimentin (mouse)	Forward: CGGCTGCGAGAGAAATTGC
	Reverse: CCACTTTCCGTTCAAGGTCAAG
Fibronectin (mouse)	Forward: GTGACACTTATGAGCGCCCTA
	Reverse: CCACTTGTCGCCAATCTTGTA
α-SMA (mouse)	Forward: GGACGTACAACTGGTATTGTGC
	Reverse: TCGGCAGTAGTCACGAAGGA
